# Oscillations in pedunculopontine nucleus in Parkinson’s disease and its relationship with deep brain stimulation

**DOI:** 10.3389/fncir.2015.00047

**Published:** 2015-09-02

**Authors:** Min Li, Wangming Zhang

**Affiliations:** The National Key Clinic Specialty, Guangdong Provincial Key Laboratory on Brain Function Repair and Regeneration, The Engineering Technology Research Center of Education Ministry of China, Department of Neurosurgery, Zhujiang Hospital, Southern Medical UniversityGuangzhou, China

**Keywords:** pedunculopontine nucleus, oscillations, Parkinson’s disease, local field potentials, deep brain stimulation

## Abstract

The recent development of deep brain stimulation (DBS) of the pedunculopontine nucleus (PPN) for the treatment of parkinsonian patients, particularly those in advanced stages with axial symptoms, has ignited interest into the study of this brain nucleus. In contrast to the extensively studied alterations of neural activity that occur in the basal ganglia in Parkinson’s disease (PD), our understanding of the activity of the PPN remains insufficient. In recent years, however, a series of studies recording oscillatory activity in the PPN of parkinsonian patients have made important findings. Here, we briefly review recent studies that explore the different kinds of oscillations observed in the PPN of parkinsonian patients, and how they underlie the pathophysiology of PD and the efficacy of PPN-DBS in these disorders.

## Introduction

The use of single unit or action potential recordings to examine increases or decreases in neural firing does not fully capture the dysfunction of the basal ganglia in Parkinson’s disease (PD). In recent years, the use of local field potential (LFP) recordings has made a resurgence in the investigation of the pathophysiology of Parkinsonian and other movement disorders. LFP, similar to EEG, is a kind of oscillatory activities, which is classified by the oscillatory frequency. Generally, LFP is divided into five bands: delta band (1–3 Hz), theta band (4–7 Hz), alpha band (8–13 Hz), beta band (14–30 Hz), and gamma band (>30 Hz). The boundaries of these bands may be a little different in studies. Patients’ LFP is recorded through the electrode used for deep brain stimulation (DBS) after electrode implantation and before connection to the stimulator. In animals, special metal microelectrodes are planted into brain to record LFP. Several bands of oscillatory activities have been detected in the cortico-basal ganglia circuits, including in the subthalamic nucleus (STN) and the internal segment of the globus pallidus (Brown, 2003; Boraud et al., 2005; Gatev et al., 2006; Uhlhaas and Singer, 2006; Eusebio and Brown, 2007). Among these, aberrant beta band oscillations have been deemed to be one of the most important findings in the brain of PD patients (Levy et al., 2002; Fogelson et al., 2006; Weinberger et al., 2006; Chen et al., 2007; Hammond et al., 2007). These beta band abnormalities are believed to have anti-kinetic effects, and are likely to be responsible for some PD symptoms (Brown et al., 2001; Fogelson et al., 2006).

In advanced stages of PD, axial symptoms such as severe gait and postural impairments that are not ameliorated by levodopa and STN stimulation present difficult obstacles for many patients. To relieve these symptoms, targeting the pedunculopontine nucleus (PPN) with DBS holds some promise (Moro et al., 2010; Thevathasan et al., 2011; Wilcox et al., 2011; Morita et al., 2014). Although the PPN generally is not considered part of the cortico-basal ganglia loop, there are extensive reciprocal connections between the PPN and the basal ganglia. Therefore, the question arises of whether the PPN may exhibit the same oscillatory activity as the basal ganglia in PD. Also, what is the relationship between PPN oscillations and the pathophysiology of PD? How are PPN oscillations altered with DBS treatment? These are the questions that will be discussed in this review.

## Overview of Oscillations in the PPN

Activity in the alpha frequency has been the most noticeable oscillatory activity recorded in the PPN, first recorded in parkinsonian patients by Androulidakis et al. (2008a,b). Although alpha band oscillations have drawn less attention in studies of the basal ganglia, these activities likely play a pivotal role in the function of the PPN. For example, alpha band power increases significantly following treatment with levodopa (Androulidakis et al., 2008b; Fraix et al., 2013) and correlates with gait performance in PD patients (Thevathasan et al., 2012; Tattersall et al., 2014). These studies suggest that activity in the alpha band plays a physiological function in the PPN, and is pathologically attenuated in PD.

Unlike findings in the cortical-basal ganglia loop, there currently exists no consensus as to the importance of beta band PPN activity in PD. A number of studies have shown that dopaminergic medication has a suppressive effect upon beta activity in the PPN, implying that this activity may contribute to akinesia in PD (Thevathasan et al., 2012; Fraix et al., 2013). Other studies have found quite the contrary, however. For example, beta oscillations in the PPN were shown to decrease in the absence of medication, but increase in the presence of medication, in patients making voluntary movements. In this study, beta coherence between the midline prefrontal region and the PPN was only found in the medicated state (Tsang et al., 2010). Thus, beta rhythms may have a different functional significance in the PPN compared with the basal ganglia, in that these oscillations could be pro-kinetic.

**Figure 1:**
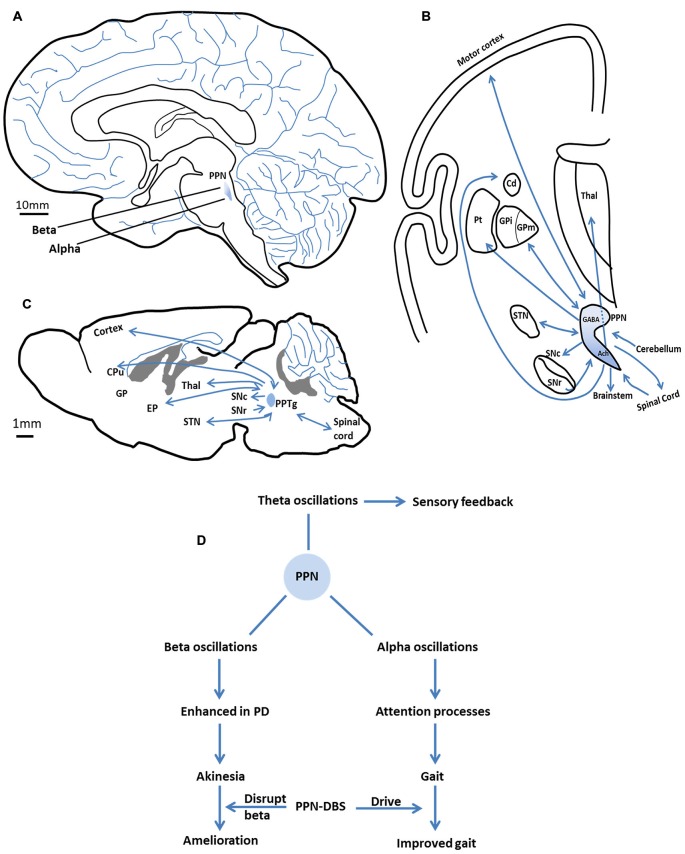
**The anatomy (location and connections) of the pedunculopontine nucleus (PPN) and the major oscillations in the PPN and their functional characteristics. (A)** The location of the PPN in the human brain. Alpha oscillations mainly present in the caudal subregion of the PPN, beta oscillations predominantly in the rostral subregion. **(B)** The major efferent and afferent pathways of the PPN to the basal ganglia and other motor structures in the human brain. Neurons in the rostral subregion mainly express GABA, while neurons in the caudal subregion are primarily cholinergic. Adapted from Jenkinson et al. (2009). **(C)** The major efferent and afferent pathways of the PPTg to the basal ganglia and other motor structures in the rat brain. **(D)** The major oscillations in the PPN and their functional characteristics. Alpha band promote gait performance by modulating attention processes. Enhanced beta band may result in akinesia. PPN-DBS may affect these two bands to improve symptoms. Theta band is involved in sensory feedback. SNc, substantia nigra compacta; SNr, substantia nigra reticular; GPi/EP, internal globus pallidus; GPm, medial pallidum; STN, subthalamic nucleus; Cd/CPu, caudate nucleus and putamen; Thal, thalamus; Pt, paratenial nucleus; GABA, gamma aminobutyric acid; Ach, acetylcholine.

Topological differences appear to exist between the alpha and beta oscillations of the caudal vs. the rostral PPN. In parkinsonian patients, alpha oscillations represented the main frequency in the caudal subregion of the PPN, while beta oscillations predominate in the rostral subregion (Figure 1A; Weinberger et al., 2008; Thevathasan et al., 2012; Tattersall et al., 2014).

Theta and gamma band oscillations have also been recorded in the PPN (Shimamoto et al., 2010; Tsang et al., 2010; Fraix et al., 2013; Valencia et al., 2014; Lau et al., 2015). In the basal ganglia, theta activity has been suggested to underlie parkinsonian tremor, which appears and disappears in synchrony with muscle tremor (Stein and Bar-Gad, 2013). In the PPN, theta oscillations may be involved in the feedback of sensory information between the PPN and the sensorimotor cortices (will be discussed below; Tsang et al., 2010). Gamma oscillations were first reported in the PPN of parkinsonian patients by Fraix et al., who proposed that their functional impact might be similar to beta oscillations (Fraix et al., 2013). This certainly requires further investigation. Table 1 shows different studies looking at oscillatory activity in the PPN. The major oscillations in the PPN and their functional characteristics are summarized in Figure 1D and will be discussed below.

**Table 1:** **Different studies looking at oscillatory activity in the PPN**.

Study	Subjects	Frequency band	Findings or speculations
Androulidakis et al. (2008a)	1 patient	Alpha (7–11 Hz)	Play a physiological role
Androulidakis et al. (2008b)	6 patients	Alpha (7–11 Hz)	Play a physiological role
			Related to attentional processes	
Weinberger et al. (2008)	7 patients	Beta (15–30 Hz)	Help to guide electrode implantation
Tsang et al. (2010)	7 patients	Theta (6–10 Hz)	Involved in sensory feedback
		Beta (14–30 Hz)	May be pro-kinetic
Thevathasan et al. (2012)	7 patients	Alpha (7–10 Hz)	Correlate with gait performance
			Suppress task irrelevant distraction
			Mainly in the caudal-PPN
		Beta (17.3–28.5 Hz)	Mainly in the rostral-PPN
Fraix et al. (2013)	7 patients	Alpha (5–12 Hz)	Pro-kinetic
		Beta (13–35 Hz)	Anti-kinetic
		Gamma (>35 Hz)	Anti-kinetic
Tattersall et al. (2014)	11 patients	Alpha (6–12 Hz)	Mainly in the caudal-PPN
		Beta (12–30 Hz)	Mainly in the rostral-PPN
Lau et al. (2015)	6 patients	Alpha (8–13 Hz)	Spatially localized within PPN
		Beta (13–30 Hz)	Spatially localized within PPN
Valencia et al. (2014)	Rats	Alpha (8–12 Hz)	Altered connectivity between motor cortex and the PPN
		Gamma (30–70 Hz)	

## Implications of PPN Oscillations for PD

Alpha band power has been correlated with gait performance in parkinsonian patients, and oscillations at this frequency are increased after application of levodopa (Thevathasan et al., 2012; Fraix et al., 2013). Given this, one may wonder how alpha activity in the PPN might relate to the amelioration of axial symptoms. Alpha activity is considered to play a vital part in attention and in the allocation of processing resources in the brain (Klimesch, 1999; Palva and Palva, 2007). Increases in alpha activity may indicate the suppression of a distracting or task-irrelevant process, concomitant with the increased ability to focus attention on an important task (Ward, 2003; Jensen and Mazaheri, 2010). For example, alpha power increases with memory load during memory tasks, reflecting the individual’s efforts to suppress distraction (Jensen et al., 2002). In the motor system, alpha activity is associated with the suppression of irrelevant processes in order to allow the smooth execution of motor programs (Pfurtscheller and Neuper, 1994; Suffczynski et al., 2001). Impairments in attentional function are common in parkinsonian patients (Wu and Hallett, 2008). Moreover, patients with axial symptoms (for example, gait freezing) are found to suffer more serious attentional impairments than those without Amboni et al. (2008) and Yogev-Seligmann et al. (2008). Thus, gait freezing is thought to be related to attentional deficits (Giladi and Hausdorff, 2006; Okuma, 2006). The results of a recent study on PPN-DBS of PD patients suggested that attentional augmentation can be “one possible mechanism to improve motor action and gait in patients with parkinsonian disorders” (Fischer et al., 2015). As part of the reticular activating system, the PPN could modulate attention via alpha oscillations to facilitate gait performance (Figure 1D).

Alpha oscillations have been recorded in a relatively caudal region of the PPN (Thevathasan et al., 2012), a region shown by animal studies to be rich in cholinergic neurons (Figures 1A,B; Martinez-Gonzalez et al., 2011). These neurons project widely, ranging from the cortex to locomotor centers (Figures 1B,C; Skinner et al., 1990; Mena-Segovia et al., 2008). Loss of cholinergic neurons in the PPN has been observed in PD, and this is thought to be associated with the gait and postural disorders of parkinsonian patients (Karachi et al., 2010; Grabli et al., 2013). Patients with a less severe loss of PPN cholinergic neurons may have better clinical outcomes with PPN-DBS therapy (Lau et al., 2015). Presumably, the dysfunction of cholinergic neurons might reduce the positive effects of PPN alpha activity in the modulation of attention processes, thereby leading to gait problems.

Theta rhythms in the PPN are hypothesized to be involved in the functional impact of sensory feedback, and not with other functions ascribed to the alpha band (Tsang et al., 2010). Based on animal studies, the PPN may be particularly involved in the sorting of sensory information for movement planning and performance (Winn, 2006). Studies of the phase relationship in theta band coherence between the cortex and PPN reveal that the sensorimotor cortex is temporally ahead of the PPN in motor planning, but is later in motor performing, suggesting that the cortex may drive the PPN in the phase of planning, then sensory information fed back to the cortex via the PPN to facilitate motor performance (Tsang et al., 2010). Coherence in theta activity has been observed in other related encephalic regions, such as the ipsilateral sensorimotor cortex and the ventral thalamus, areas that are suggested to form a system connecting related brain areas for the purpose of continuously monitoring sensory information (Nicolelis et al., 1995; Marsden et al., 2000). Thus, theta activity in the PPN may indicate that this region is part of this sensory feedback system (Figure 1D).

Within the PPN, neurons in the rostral subregion mainly express gamma aminobutyric acid (GABA) and are inhibitory, whereas neurons in the caudal subregion are primarily glutamatergic and cholinergic (Martinez-Gonzalez et al., 2011). These two regions have distinct projection patterns that connect them to disparate brain structures. Robust interconnections exist between neurons in the rostral PPN and the basal ganglia (Mena-Segovia et al., 2004; Martinez-Gonzalez et al., 2011), whereas caudal PPN neurons mainly connect with cortical and locomotor centers such as the gait generator in the spinal cord (Figure 1B; Skinner et al., 1990; Mena-Segovia et al., 2008). This neuronal heterogeneity in the PPN likely underlies the differences in oscillatory activity between PPN subregions. For example, the beta activity recorded in the rostral subregion of the PPN is consistent with reciprocal connections to the basal ganglia, which exhibit excessive beta activity in PD. In the caudal PPN, alpha activity appears to be in coherence with cortical activity and relevant to locomotion.

The oscillatory topography discussed above suggests that the depth of recording electrodes along the rostro-caudal axis will have a significant effect on the type of dominant oscillatory patterns recorded in the PPN. This is one explanation for the discrepancy between studies that have recorded different oscillations in parkinsonian patients, which could lead to inconsistent interpretations of beta activity. The differing views regarding the importance of beta band oscillations in the PPN, however, likely also originate from additional factors such as small sample size, patient heterogeneity, and the diversity of analysis methods. According to animal data, increased beta oscillatory activity in the basal ganglia can be transmitted downstream to the PPN (Aravamuthan et al., 2008). Accordingly, from the current literature, it seems likely that beta activity in the human PPN, as in the basal ganglia, could contribute to akinesia in PD (Figure 1D), but more studies are needed.

## PPN Oscillations and PPN-DBS

Although DBS has been used for decades for the treatment of PD and other movement disorders, the precise mechanisms underlying the efficacy of this therapy remain unclear. One speculation is that high frequency DBS of the STN inhibits neurons by focal depolarization, thereby blockading abnormal discharges from the basal ganglia (Filali et al., 2004; Welter et al., 2004; Chang et al., 2008). While this may be consistent with the classical Albin/Delong model (A widely accepted model of basal ganglia function based on the dominant anatomical connections of basal ganglia nuclei and their neurochemistry; Albin et al., 1989; DeLong, 1990), some doubt remains. For example, the electrical pulses used in DBS are thought to be too brief to blockade basal ganglia activity. Additionally, the long term suppression of basal ganglia activity should lead to the death of neurons and increased gliosis, yet these phenomena have not been observed in autopsies of DBS patients (Burbaud et al., 2002). A blockading effect is unlikely to be the mechanism underlying the efficacy of DBS in the PPN, since the low frequency stimulation used in this paradigm would not produce inhibitory effects.

Rather, it is possible that DBS of the PPN directly drives its activity to benefit parkinsonian states. The low frequency stimulus is the most effective for symptom relief in PPN-DBS treatment (Mazzone et al., 2005), and this paradigm is generally considered to increase neuronal activity (Aravamuthan et al., 2008). The power of alpha oscillations recorded in the PPN of parkinsonian patients indicates that this activity may have a physiological function, since it is pathologically attenuated in PD. Therefore, low frequency stimulation of the PPN may drive the neurons and simulate the inherent alpha activity. This concept is supported by the interesting finding that parkinsonian patients who underwent PPN-DBS obtained the best therapeutic effect when the stimulation electrode was placed in the site of maximal alpha activity, namely, the caudal PPN (Thevathasan et al., 2012). Furthermore, non-human primate research suggests that the PPN is over-inhibited in parkinsonian states (Matsumura and Kojima, 2001; Nandi et al., 2002). Accordingly, the ability of DBS to drive the PPN is a likely mechanism for its therapeutic effects in PD (Figure 1D).

From recent studies, based upon recordings of pathological oscillatory activity (beta oscillations) in the cortical-basal ganglia loop in parkinsonian patients, it has been suggested that DBS may exert beneficial effects via the disruption of this aberrant activity. In the PPN, it is possible that DBS may exert its positive effects in this manner (Figure 1D). It has been suggested that pathological beta oscillations recorded in the PPN could have been transmitted downstream from the basal ganglia. Moreover, low frequency PPN-DBS was shown to reduce beta frequency activity in the STN of 6-OHDA lesioned rats (Alam et al., 2012).

Another mechanism, one that involves spike timing, could underlie the therapeutic effects of PPN-DBS. Studies of the relationship between PPN spiking and LFP activity found that the PPN spike timing changed significantly following 6-OHDA lesioning in rats. In intact rats, PPN firing tended to occur at the troughs of LFP oscillations, and this neuronal firing became peak-locked in the lesioned rats (Aravamuthan et al., 2008). Based on this evidence, it is possible that PPN-DBS affects PPN spike-timing relationship to normalize activity.

As mentioned, alpha and beta oscillations in the PPN are spatially localized (Thevathasan et al., 2012; Lau et al., 2015); thus this topographical arrangement can provide a guide for DBS electrode implantation. The finding that stimulation at the position of maximal alpha activity results in the most effective amelioration of symptoms suggests that the site of stimulation in the PPN is a crucial factor affecting therapeutic outcomes, one that merits more attention. Along these lines, a recent study on parkinsonian rats found distinct effects of the pedunculopontine tegmental nucleus (PPTg, the equivalent to the human PPN) DBS at different sites of stimulation, and emphasized “the critical importance of intra-PPTg DBS location” (Gut and Winn, 2015). The PPN is a heterogeneous structure in the brainstem that presents with an uncertainty boundary, and which is involved in multiple functions including cortical arousal, the control of behavioral processing, and locomotion (Ros et al., 2010; Benarroch, 2013). Different subregions in the PPN may have different functions (Martinez-Gonzalez et al., 2011). With regard to motor function, some processes may be closely associated with the cortico-basal ganglia loop, while others have connections with locomotion centers such as the gait generator in the spinal cord. The function of each subregion as well as their interconnections merits further study.

## Conclusion

Studies that record oscillatory activity in the PPN have provided a great deal of information regarding the pathophysiology of PD and the mechanisms underlying the effects of PPN-DBS. Alpha activity is considered to be important in the PPN, where it may modulate attentional processes to improve gait performance in parkinsonian patients. DBS of the PPN could alleviate gait symptoms by driving PPN neurons and thereby affecting alpha oscillations. Nevertheless, PPN-DBS therapy remains experimental, and only a small number of patients are available to study. Large multicenter clinical trials are needed to best study the efficacy of PPN-DBS in PD. At the same time, more animal research would greatly supplement human studies by more mechanistic examination of this enigmatic brain region.

## Conflict of Interest Statement

The authors declare that the research was conducted in the absence of any commercial or financial relationships that could be construed as a potential conflict of interest.

## References

[B1] Alam M. Heissler H. E. Schwabe K. Krauss J. K. (2012). Deep brain stimulation of the pedunculopontine tegmental nucleus modulates neuronal hyperactivity and enhanced beta oscillatory activity of the subthalamic nucleus in the rat 6-hydroxydopamine model. Exp. Neurol. 233, 233–242. 10.1016/j.expneurol.2011.10.006 22036687

[B2] Albin R. L. Young A. B. Penney J. B. (1989). The functional anatomy of basal ganglia disorders. Trends Neurosci. 12, 366–375. 10.1016/0166-2236(89)90074-x 2479133

[B3] Amboni M. Cozzolino A. Longo K. Picillo M. Barone P. (2008). Freezing of gait and executive functions in patients with Parkinson’s disease. Mov. Disord. 23, 395–400. 10.1002/mds.21850 18067193

[B4] Androulidakis A. G. Khan S. Litvak V. Pleydell-Pearce C. W. Brown P. Gill S. S. (2008a). Local field potential recordings from the pedunculopontine nucleus in a Parkinsonian patient. Neuroreport 19, 59–62. 10.1097/wnr.0b013e3282f2e2d1 18281893

[B5] Androulidakis A. G. Mazzone P. Litvak V. Penny W. Dileone M. Gaynor L. M. (2008b). Oscillatory activity in the pedunculopontine area of patients with Parkinson’s disease. Exp. Neurol. 211, 59–66. 10.1016/j.expneurol.2008.01.002 18282571

[B6] Aravamuthan B. R. Bergstrom D. A. French R. A. Taylor J. J. Parr-Brownlie L. C. Walters J. R. (2008). Altered neuronal activity relationships between the pedunculopontine nucleus and motor cortex in a rodent model of Parkinson’s disease. Exp. Neurol. 213, 268–280. 10.1016/j.expneurol.2008.05.023 18601924 PMC4318559

[B7] Benarroch E. E. (2013). Pedunculopontine nucleus: functional organization and clinical implications. Neurology 80, 1148–1155. 10.1212/wnl.0b013e3182886a76 23509047

[B8] Boraud T. Brown P. Goldberg J. Graybiel A. Magill P. (2005). “Oscillations in the basal ganglia: the good, the bad and the unexpected,” in The Basal Ganglia VIII, eds Bolam J. P. Ingham, C. Magill P. (New York: Springer Science and Business Media), 1–24.

[B9] Brown P. (2003). Oscillatory nature of human basal ganglia activity: relationship to the pathophysiology of Parkinson’s disease. Mov. Disord. 18, 357–363. 10.1002/mds.10358 12671940

[B10] Brown P. Oliviero A. Mazzone P. Insola A. Tonali P. Di Lazzaro V. (2001). Dopamine dependency of oscillations between subthalamic nucleus and pallidum in Parkinson’s disease. J. Neurosci. 21, 1033–1038. 11157088 10.1523/JNEUROSCI.21-03-01033.2001 PMC6762327

[B11] Burbaud P. Vital A. Rougier A. Bouillot S. Guehl D. Cuny E.. (2002). Minimal tissue damage after stimulation of the motor thalamus in a case of chorea-acanthocytosis. Neurology 59, 1982–1984. 10.1212/01.wnl.0000038389.30437.1e 12499498

[B12] Chang J. Y. Shi L. H. Luo F. Zhang W. M. Woodward D. J. (2008). Studies of the neural mechanisms of deep brain stimulation in rodent models of Parkinson’s disease. Neurosci. Biobehav. Rev. 32, 352–366. 10.1016/j.neubiorev.2007.09.002 18035416

[B13] Chen C. C. Litvak V. Gilbertson T. Kühn A. Lu C. S. Lee S. T.. (2007). Excessive synchronization of basal ganglia neurons at 20 Hz slows movement in Parkinson’s disease. Exp. Neurol. 205, 214–221. 10.1016/j.expneurol.2007.04.003 17335810

[B14] DeLong M. R. (1990). Primate models of movement disorders of basal ganglia origin. Trends Neurosci. 13, 281–285. 10.1016/0166-2236(90)90110-v 1695404

[B15] Eusebio A. Brown P. (2007). Oscillatory activity in the basal ganglia. Parkinsonism Relat. Disord. 13(Suppl. 3), S434–S436. 10.1016/S1353-8020(08)70044-0 18267278

[B16] Filali M. Hutchison W. D. Palter V. N. Lozano A. M. Dostrovsky J. O. (2004). Stimulation-induced inhibition of neuronal firing in human subthalamic nucleus. Exp. Brain Res. 156, 274–281. 10.1007/s00221-003-1784-y 14745464

[B17] Fischer J. Schwiecker K. Bittner V. Heinze H. J. Voges J. Galazky I.. (2015). Modulation of attentional processing by deep brain stimulation of the pedunculopontine nucleus region in patients with Parkinsonian disorders. Neuropsychology 29, 632–637. 10.1037/neu0000179 25643214

[B18] Fogelson N. Williams D. Tijssen M. van Bruggen G. Speelman H. Brown P. (2006). Different functional loops between cerebral cortex and the subthalmic area in Parkinson’s disease. Cereb. Cortex 16, 64–75. 10.1093/cercor/bhi084 15829734

[B19] Fraix V. Bastin J. David O. Goetz L. Ferraye M. Benabid A. L.. (2013). Pedunculopontine nucleus area oscillations during stance, stepping and freezing in Parkinson’s disease. PLoS One 8:e83919. 10.1371/journal.pone.0083919 24386308 PMC3875496

[B20] Gatev P. Darbin O. Wichmann T. (2006). Oscillations in the basal ganglia under normal conditions and in movement disorders. Mov. Disord. 21, 1566–1577. 10.1002/mds.21033 16830313

[B21] Giladi N. Hausdorff J. M. (2006). The role of mental function in the pathogenesis of freezing of gait in Parkinson’s disease. J. Neurol. Sci. 248, 173–176. 10.1016/j.jns.2006.05.015 16780886

[B22] Grabli D. Karachi C. Folgoas E. Monfort M. Tande D. Clark S.. (2013). Gait disorders in parkinsonian monkeys with pedunculopontine nucleus lesions: a tale of two systems. J. Neurosci. 33, 11986–11993. 10.1523/jneurosci.1568-13.2013 23864685 PMC6794061

[B23] Gut N. K. Winn P. (2015). Deep brain stimulation of different pedunculopontine targets in a novel rodent model of parkinsonism. J. Neurosci. 35, 4792–4803. 10.1523/JNEUROSCI.3646-14.2015 25810510 PMC4389588

[B24] Hammond C. Bergman H. Brown P. (2007). Pathological synchronization in Parkinson’s disease: networks, models and treatments. Trends Neurosci. 30, 357–364. 10.1016/j.tins.2007.05.004 17532060

[B25] Jenkinson N. Nandi D. Muthusamy K. Ray N. J. Gregory R. Stein J. F.. (2009). Anatomy, physiology and pathophysiology of the pedunculopontine nucleus. Mov. Disord. 24, 319–328. 10.1002/mds.22189 19097193

[B26] Jensen O. Gelfand J. Kounios J. Lisman J. E. (2002). Oscillations in the alpha band (9–12 Hz) increase with memory load during retention in a short-term memory task. Cereb. Cortex 12, 877–882. 10.1093/cercor/12.8.877 12122036

[B27] Jensen O. Mazaheri A. (2010). Shaping functional architecture by oscillatory alpha activity: gating by inhibition. Front. Hum. Neurosci. 4:186. 10.3389/fnhum.2010.00186 21119777 PMC2990626

[B28] Karachi C. Grabli D. Bernard F. A. Tande D. Wattiez N. Belaid H.. (2010). Cholinergic mesencephalic neurons are involved in gait and postural disorders in Parkinson disease. J. Clin. Invest. 120, 2745–2754. 10.1172/JCI42642 20628197 PMC2912198

[B29] Klimesch W. (1999). EEG alpha and theta oscillations reflect cognitive and memory performance: a review and analysis. Brain Res. Brain Res. Rev. 29, 169–195. 10.1016/s0165-0173(98)00056-3 10209231

[B30] Lau B. Welter M. L. Belaid H. Fernandez Vidal S. Bardinet E. Grabli D.. (2015). The integrative role of the pedunculopontine nucleus in human gait. Brain 138, 1284–1296. 10.1093/brain/awv047 25765327 PMC5963406

[B31] Levy R. Ashby P. Hutchison W. D. Lang A. E. Lozano A. M. Dostrovsky J. O. (2002). Dependence of subthalamic nucleus oscillations on movement and dopamine in Parkinson’s disease. Brain 125, 1196–1209. 10.1093/brain/awf128 12023310

[B32] Marsden J. F. Ashby P. Limousin-Dowsey P. Rothwell J. C. Brown P. (2000). Coherence between cerebellar thalamus, cortex and muscle in man: cerebellar thalamus interactions. Brain 123, 1459–1470. 10.1093/brain/123.7.1459 10869057

[B33] Martinez-Gonzalez C. Bolam J. P. Mena-Segovia J. (2011). Topographical organization of the pedunculopontine nucleus. Front. Neuroanat. 5:22. 10.3389/fnana.2011.00022 21503154 PMC3074429

[B34] Matsumura M. Kojima J. (2001). The role of the pedunculopontine tegmental nucleus in experimental parkinsonism in primates. Stereotact. Funct. Neurosurg. 77, 108–115. 10.1159/000064614 12378066

[B35] Mazzone P. Lozano A. Stanzione P. Galati S. Scarnati E. Peppe A.. (2005). Implantation of human pedunculopontine nucleus: a safe and clinically relevant target in Parkinson’s disease. Neuroreport 16, 1877–1881. 10.1097/01.wnr.0000187629.38010.12 16272871

[B36] Mena-Segovia J. Bolam J. P. Magill P. J. (2004). Pedunculopontine nucleus and basal ganglia: distant relatives or part of the same family? Trends Neurosci. 27, 585–588. 10.1016/j.tins.2004.07.009 15374668

[B37] Mena-Segovia J. Sims H. M. Magill P. J. Bolam J. P. (2008). Cholinergic brainstem neurons modulate cortical gamma activity during slow oscillations. J. Physiol. 586, 2947–2960. 10.1113/jphysiol.2008.153874 18440991 PMC2517196

[B38] Morita H. Hass C. J. Moro E. Sudhyadhom A. Kumar R. Okun M. S. (2014). Pedunculopontine nucleus stimulation: where are we now and what needs to be done to move the field forward? Front. Neurol. 5:243. 10.3389/fneur.2014.00243 25538673 PMC4255598

[B39] Moro E. Hamani C. Poon Y. Y. Al-Khairallah T. Dostrovsky J. O. Hutchison W. D.. (2010). Unilateral pedunculopontine stimulation improves falls in Parkinson’s disease. Brain 133, 215–224. 10.1093/brain/awp261 19846583

[B40] Nandi D. Aziz T. Z. Giladi N. Winter J. Stein J. F. (2002). Reversal of akinesia in experimental parkinsonism by GABA antagonist microinjections in the pedunculopontine nucleus. Brain 125, 2418–2430. 10.1093/brain/awf259 12390969

[B41] Nicolelis M. A. Baccala L. A. Lin R. C. Chapin J. K. (1995). Sensorimotor encoding by synchronous neural ensemble activity at multiple levels of the somatosensory system. Science 268, 1353–1358. 10.1126/science.7761855 7761855

[B42] Okuma Y. (2006). Freezing of gait in Parkinson’s disease. J. Neurol. 253, VII27–VII32. 10.1016/j.gaitpost.2014.09.021 17131225

[B43] Palva S. Palva J. M. (2007). New vistas for alpha-frequency band oscillations. Trends Neurosci. 30, 150–158. 10.1016/j.tins.2007.02.001 17307258

[B44] Pfurtscheller G. Neuper C. (1994). Event-related synchronization of mu rhythm in the EEG over the cortical hand area in man. Neurosci. Lett. 174, 93–96. 10.1016/0304-3940(94)90127-9 7970165

[B45] Ros H. Magill P. J. Moss J. Bolam J. P. Mena-Segovia J. (2010). Distinct types of non-cholinergic pedunculopontine neurons are differentially modulated during global brain states. Neuroscience 170, 78–91. 10.1016/j.neuroscience.2010.06.068 20603194 PMC4242969

[B46] Shimamoto S. A. Larson P. S. Ostrem J. L. Glass G. A. Turner R. S. Starr P. A. (2010). Physiological identification of the human pedunculopontine nucleus. J. Neurol. Neurosurg. Psychiatry 81, 80–86. 10.1136/jnnp.2009.179069 19828478 PMC3806635

[B47] Skinner R. D. Kinjo N. Henderson V. Garcia-Rill E. (1990). Locomotor projections from the pedunculopontine nucleus to the spinal cord. Neuroreport 1, 183–186. 10.1097/00001756-199011000-00001 2129877

[B48] Stein E. Bar-Gad I. (2013). beta oscillations in the cortico-basal ganglia loop during parkinsonism. Exp. Neurol. 245, 52–59. 10.1016/j.expneurol.2012.07.023 22921537

[B49] Suffczynski P. Kalitzin S. Pfurtscheller G. Lopes da Silva F. H. (2001). Computational model of thalamo-cortical networks: dynamical control of alpha rhythms in relation to focal attention. Int. J. Psychophysiol. 43, 25–40. 10.1016/s0167-8760(01)00177-5 11742683

[B50] Tattersall T. L. Stratton P. G. Coyne T. J. Cook R. Silberstein P. Silburn P. A.. (2014). Imagined gait modulates neuronal network dynamics in the human pedunculopontine nucleus. Nat. Neurosci. 17, 449–454. 10.1038/nn.3642 24487235

[B51] Thevathasan W. Coyne T. J. Hyam J. A. Kerr G. Jenkinson N. Aziz T. Z.. (2011). Pedunculopontine nucleus stimulation improves gait freezing in Parkinson disease. Neurosurgery 69, 1248–1253; discussion 1254. 10.1227/neu.0b013e31822b6f71 21725254

[B52] Thevathasan W. Pogosyan A. Hyam J. A. Jenkinson N. Foltynie T. Limousin P.. (2012). Alpha oscillations in the pedunculopontine nucleus correlate with gait performance in parkinsonism. Brain 135, 148–160. 10.1093/brain/awr315 22232591 PMC3267984

[B53] Tsang E. W. Hamani C. Moro E. Mazzella F. Poon Y. Y. Lozano A. M.. (2010). Involvement of the human pedunculopontine nucleus region in voluntary movements. Neurology 75, 950–959. 10.1212/WNL.0b013e3181f25b35 20702790 PMC2942031

[B54] Uhlhaas P. J. Singer W. (2006). Neural synchrony in brain disorders: relevance for cognitive dysfunctions and pathophysiology. Neuron 52, 155–168. 10.1016/j.neuron.2006.09.020 17015233

[B55] Valencia M. Chavez M. Artieda J. Bolam J. P. Mena-Segovia J. (2014). Abnormal functional connectivity between motor cortex and pedunculopontine nucleus following chronic dopamine depletion. J. Neurophysiol. 111, 434–440. 10.1152/jn.00555.2013 24174651 PMC3921386

[B56] Ward L. M. (2003). Synchronous neural oscillations and cognitive processes. Trends Cogn. Sci. 7, 553–559. 10.1016/j.tics.2003.10.012 14643372

[B57] Weinberger M. Hamani C. Hutchison W. D. Moro E. Lozano A. M. Dostrovsky J. O. (2008). Pedunculopontine nucleus microelectrode recordings in movement disorder patients. Exp. Brain Res. 188, 165–174. 10.1007/s00221-008-1349-1 18347783

[B58] Weinberger M. Mahant N. Hutchison W. D. Lozano A. M. Moro E. Hodaie M.. (2006). Beta oscillatory activity in the subthalamic nucleus and its relation to dopaminergic response in Parkinson’s disease. J. Neurophysiol. 96, 3248–3256. 10.1152/jn.00697.2006 17005611

[B59] Welter M. L. Houeto J. L. Bonnet A. M. Bejjani P. B. Mesnage V. Dormont D.. (2004). Effects of high-frequency stimulation on subthalamic neuronal activity in parkinsonian patients. Arch. Neurol. 61, 89–96. 10.1001/archneur.61.1.89 14732625

[B60] Wilcox R. A. Cole M. H. Wong D. Coyne T. Silburn P. Kerr G. (2011). Pedunculopontine nucleus deep brain stimulation produces sustained improvement in primary progressive freezing of gait. J. Neurol. Neurosurg. Psychiatry 82, 1256–1259. 10.1136/jnnp.2010.213462 20971757

[B61] Winn P. (2006). How best to consider the structure and function of the pedunculopontine tegmental nucleus: evidence from animal studies. J. Neurol. Sci. 248, 234–250. 10.1016/j.jns.2006.05.036 16765383

[B62] Wu T. Hallett M. (2008). Neural correlates of dual task performance in patients with Parkinson’s disease. J. Neurol. Neurosurg. Psychiatry 79, 760–766. 10.1136/jnnp.2007.126599 18006652

[B63] Yogev-Seligmann G. Hausdorff J. M. Giladi N. (2008). The role of executive function and attention in gait. Mov. Disord. 23, 329–342. 10.1002/mds.21720 18058946 PMC2535903

